# Primary care consultation modality and acute mental health service use in adults

**DOI:** 10.1038/s44220-026-00605-9

**Published:** 2026-03-17

**Authors:** Liliana Hidalgo-Padilla, Eoin Gogarty, Reese Sarkodie, Fiona Gaughran, Robert Stewart, Mariana Pinto da Costa

**Affiliations:** 1 Institute of Psychiatry, Psychology & Neuroscience, King’s College London, London, UK; 2 South London and Maudsley NHS Foundation Trust, London, UK

**Keywords:** Health services, Public health

## Abstract

The adoption of remote consultations following the COVID-19 pandemic raised questions about the impact on patient outcomes. Here we assess the relationship between the proportion of remote consultations in primary care and subsequent acute mental health service use, specifically emergency contacts with mental health liaison teams, psychiatric hospital admissions, inpatient bed-days and compulsory admissions under the Mental Health Act. We conducted a retrospective cohort study of patients diagnosed with depression, anxiety or severe mental illness using the Clinical Record Interactive Search platform, which accesses pseudonymized electronic patient records from a large London mental healthcare provider. These records were linked to primary care consultation data from the Lambeth DataNet platform. The study period covered 1 January 2019 to 31 December 2021, spanning prepandemic and pandemic phases. Associations were estimated using generalized estimating equations with a negative binomial distribution to account for overdispersion and clustering by general practice. Multiple imputation by chained equations was used to address missing data. The analytic cohort included 107,993 patients. A higher proportion of remote consultations was associated with a modest increase in the rate of emergency contacts with mental health liaison teams (incidence rate ratio (IRR) 1.04, 95% confidence interval (CI) 1.01–1.07 per 10-percentage-point increase in remote care). By contrast, no significant associations were found between consultation modality and psychiatric hospital admissions (IRR 1.03, 95% CI 1.00–1.07), inpatient bed-days (IRR 1.02, 95% CI 0.95–1.09) or compulsory admissions (IRR 1.03, 95% CI 0.99–1.07). Patients who used remote primary care consultations more frequently, particularly those delivered via telephone, had more emergency contacts with mental health liaison teams, potentially reflecting precautionary referrals or reverse causality. However, remote consulting was not associated with increased psychiatric admissions, longer hospital stays or compulsory detentions, suggesting it is not linked to clinical deterioration requiring hospitalizations.

## Abstract (web summary)

Using retrospective health-record analyses, Hidalgo-Padilla et al. evaluated whether differences in remote versus in-person primary care consultations were associated with subsequent use of acute mental health services in the UK.

## Main

Primary care is the cornerstone of health systems, as emphasized by the World Health Organization in the Alma-Ata Declaration^1^. It encompasses medical care provided by general practitioners (GPs), supported by a multidisciplinary primary care team^2^. Primary care plays a crucial role in mental health care, with approximately 90% of mental health conditions being managed within primary care settings^3^. GPs and primary care nurses provide care for individuals with mild-to-moderate mental health conditions^4^, which can help reduce the mental health treatment gap^5^. In recognition of this, the National Health Service (NHS) Mental Health Implementation Plan 2019/2020–2023/2024 committed to developing integrated models of primary and community mental health care to enhance access and improve the quality of care for people living with mental illness^6^.

Recent global epidemiological evidence indicates that anxiety disorders, major depressive disorder, schizophrenia and bipolar disorder account for the highest age-standardized disability-adjusted life years among mental disorders^7^. Given this high burden, understanding their pathways in primary care is essential. In 2016, the 12-month prevalence of depression and severe mental illness (SMI), specifically bipolar and psychotic disorders, in English primary care was 8.4% and 0.9%, respectively^8^. Within primary care registers in South London, the prevalence of ever-diagnosed conditions is 16.4% for anxiety, 17% for depression and 1.7% for SMI^9^. Regarding management, differences between these groups are marked. A study conducted in England, adjusting for practice sizes and socioeconomic deprivation indices, found that 31.1% of patients with SMI were managed exclusively in primary care over the 12-month study period, while 68.9% also had secondary care contacts^10^. By contrast, anxiety and depression disorders tend to be managed in primary care in 76.2% and 70.1% of cases, respectively^9^.

Patients requiring specialized care are referred to secondary care. In cases of mental health crises, patients can be admitted to a hospital voluntarily or under compulsory provisions, which in the UK is regulated by the Mental Health Act (MHA). Under the MHA, patients may be detained if they are in need of urgent treatment for a mental health disorder and pose a risk of harm to themselves or others^11^.

### Remote care for mental health

Remote consultations were first introduced in 2012 by the UK Department of Health and Social Care^12^. While their uptake slowly grew^13–15^, it was not until the coronavirus disease 2019 (COVID-19) pandemic that remote consultations were implemented on a national scale and at pace. GPs and other NHS clinicians had to rapidly transition from traditional face-to-face consultations to consultations delivered remotely via telephone, text, emails or video calls^16^, including mental health consultations^14,17,18^.

During the beginning of the pandemic, the number of mental health consultations in primary care for people with common mental disorders initially declined sharply. However, despite a 13% reduction in in-hours GP services for mental health conditions^19^, calls to NHS 111 and out-of-hours GP service mental health-related attendances increased by 10% and 21% compared with the previous year. In the same period and despite the decrease in consultations, studies have reported that overall GP prescriptions of antidepressants increased between 4% and 6% over previous years^20,21^.

Prior research has highlighted potential differences between remote and face-to-face consultations, including reduced opportunities for non-verbal assessment, and challenges in risk appraisal^22^. Evidence from patient experience studies also suggests that communication quality and therapeutic rapport may differ by modality^14,23,24^. Together, these factors raise the possibility that consultation modality may influence acute mental health service use, such as emergency contacts or psychiatric hospital admissions.

Given the impact of the pandemic on mental health and the widespread adoption of remote consultations, this exploratory study aims to assess the relationship between remote consultations in primary care and key acute mental health service use outcomes, including emergency contacts with mental health liaison teams, psychiatric hospital admissions, inpatient bed-days and involuntary admissions under the MHA. Although we did not specify formal hypotheses, previous literature suggesting differences in assessment and risk appraisal between remote and face-to-face consultations led us to anticipate potential differences in crisis-related outcomes depending on modality.

## Results

### Patient characteristics

**Fig. 1:**
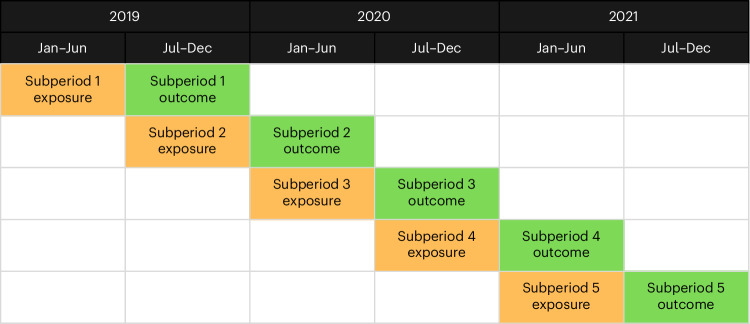
The five exposure–outcome subperiods, distributed across the full study period.

A total of 110,310 patients were recorded in Lambeth DataNet (LDN), a database with primary care consultation data from the London borough of Lambeth, as having diagnoses of depression, anxiety or SMI and consulted primary care across the exposure periods (1 January 2019 to 30 June 2021; Fig. 1). After applying exclusion criteria, 107,993 patients were included in the analytic cohort. These patients received 1,549,474 GP consultations in total throughout this period. Of these, 756,782 (48.8%) consultations were conducted face to face, 706,169 (45.6%) were remote, and 86,523 (5.6%) consultations did not have adequate data to determine their type or modality. Among the face-to-face consultations, the most common appointment types were GP surgery visits (739,642; 97.7%), walk-in clinics (5,251; 0.7%) and nursing home visits (4,064; 0.5%), whereas remote consultations were most commonly done by telephone (680,726; 96.4%), with smaller numbers by text (6,068; 0.9%), email (3,071; 0.4%) and video calls (681, 0.1%).

Four variables contained missing data: sex, ethnicity, Index of Multiple Deprivation (IMD) and consultation modality. Analysis of missingness patterns revealed systematic differences between patients with complete and incomplete records. Patients with missing ethnicity or consultation modality data tended to be younger, while certain ethnic groups tended to have more missing IMD data. In general, missing data showed substantial variation across GP practices, suggesting that missingness was partly driven by practice-level recording differences. These findings confirm that the complete-case population differed from the full study cohort, which led us to use Multiple Imputation by Chained Equations (MICE). Full missingness patterns and imputation diagnostics are provided in Supplementary Tables 1–5 and Supplementary Fig. 1.

Table 1 presents patient characteristics for the imputed study sample overall and across five exposure–outcome subperiods (Fig. 1). The cohort had a mean age of approximately 46 years and was predominantly female. Ethnic, socioeconomic and clinical history distributions were broadly stable across subperiods. The proportion of consultations conducted remotely increased over time, reflecting changes in service delivery patterns. Total consultation numbers showed a decrease at the start of the study subperiod 3, which coincides with the start of the COVID-19 pandemic, but rose in later periods. Sample characteristics for the complete-case cohort are presented in Supplementary Table 6.

**Table 1:** Patient characteristics by study subperiod

Variables	Overall	Subperiod 1	Subperiod 2	Subperiod 3	Subperiod 4	Subperiod 5
*N*	107,993	24,508	22,610	19,043	19,333	22,499
Age (years), mean (s.d.)	45.7 (18.0)	45.7 (18.2)	46.1 (18.1)	46.4 (17.8)	45.7 (17.8)	44.7 (17.8)
Sex, *n* (%)						
Male	43,465 (40.2)	9,972 (40.7)	9,276 (41.0)	7628 (40.1)	7585 (39.2)	9,004 (40.0)
Female	64,528 (59.8)	14,536 (59.3)	13,334 (59.0)	11,415 (59.9)	11,748 (60.8)	13,495 (60.0)
Ethnicity, *n* (%)						
White	49,309 (45.7)	11,314 (46.2)	10,280 (45.5)	8,555 (44.9)	8,633 (44.7)	10,527 (46.8)
Black, Black British, Caribbean or African	23,171 (21.5)	4,981 (20.3)	4,877 (21.6)	4,277 (22.5)	4,307 (22.3)	4,729 (21.0)
Asian or Asian British	7,892 (7.3)	1,834 (7.5)	1,641 (7.3)	1,396 (7.3)	1,428 (7.4)	1,593 (7.1)
Mixed or multiple ethnic groups	2,4641 (22.8)	5,770 (23.5)	5,188 (22.9)	4,261 (22.4)	4,431 (22.9)	4,991 (22.2)
Other ethnic groups	2,980 (2.8)	609 (2.5)	624 (2.8)	554 (2.9)	534 (2.8)	659 (2.9)
IMD decile, median [Q1, Q3]	4.0 [3.0, 5.0]	4.0 [3.0, 5.0]	4.0 [3.0, 5.0]	4.0 [3.0, 5.0]	4.0 [3.0, 5.0]	4.0 [3.0, 5.0]
History of anxiety, *n* (%)	54,409 (50.4)	11,874 (48.4)	11,119 (49.2)	9,632 (50.6)	9,865 (51.0)	11,919 (53.0)
History of depression, *n* (%)	86,474 (80.1)	19,896 (81.2)	18,269 (80.8)	15,283 (80.3)	15,472 (80.0)	17,554 (78.0)
History of SMI, *n* (%)	5,889 (5.5)	1,297 (5.3)	1,197 (5.3)	1,080 (5.7)	1,046 (5.4)	1,269 (5.6)
Total GP consultations, median [Q1, Q3]	3.0 [1.0, 5.0]	2.0 [1.0, 4.0]	2.0 [1.0, 5.0]	2.0 [1.0, 5.0]	3.0 [2.0, 6.0]	3.0 [2.0, 6.0]
Proportion of remote consultations (continuous), median [Q1, Q3]	0.5 [0.0, 0.7]	0.0 [0.0, 0.5]	0.0 [0.0, 0.5]	0.5 [0.0, 0.8]	0.6 [0.4, 0.9]	0.6 [0.4, 0.8]

Values are based on the imputed dataset.

The LDN dataset was linked to data from the Clinical Record Interactive Search (CRIS) system, which contains data from electronic health records of the South London and Maudsley (SLaM) NHS Trust. The linked SLaM dataset contained 10,864 emergency contacts with mental health liaison teams, 2,691 psychiatric hospital admissions, 1,848 MHA admissions and 183,286 inpatient bed-days during the study period. At the individual level, distributions were highly right skewed. Full distribution diagnostics are presented in Supplementary Table 7.

### Impact of remote consultations in acute mental health service use

Diagnostic checks confirmed that the negative binomial generalized estimating equations (GEEs) were appropriate to the data. DHARMa-derived QQ plots and residuals-versus-predicted plots showed adequate fit, with minor deviations observed only for inpatient bed-days due to the high skew of length of stay data. While the data contained a high frequency of zeros, the high dispersion ratios supported the use of the negative binomial distribution, which explicitly models this dispersion. Regarding imputation, while fraction of missing information (FMI) values indicated a moderate contribution of imputed data to uncertainty, relative increase in variance (RIV) values remained low for key exposures, suggesting that imputation did not materially destabilize the results. Full model outputs, including the quasi-likelihood under the independence model criterion (QIC) statistics, residual plots and detailed imputation diagnostics (FMI and RIV), are provided in Supplementary Tables 8–10 and Supplementary Figs. 2 and 3.

After adjusting for demographic and clinical characteristics, total number of consultations and subperiods (Table 2), the primary imputed analysis, clustering by GP practice, indicated that a higher proportion of remote consultations was associated with a small but statistically significant increase in the rate of emergency contacts with mental health liaison teams. For every 10-percentage-point increase in the proportion of remote consultations, most of which were conducted by phone, emergency contacts increased by approximately 4% (incidence rate ratio (IRR) 1.04, 95% confidence interval (CI) 1.01–1.07, *P* = 0.021). However, there was no evidence of an association between consultation modality and psychiatric hospital admissions, inpatient bed-days or MHA admissions.

**Table 2:** IRRs for emergency contacts with mental health liaison teams, psychiatric hospital admissions, inpatient bed-days and MHA admissions

Covariate	*N* of emergency contacts with mental health liaison teams	*N* of psychiatric hospital admissions	*N* of inpatient bed-days	*N* of MHA admissions
Proportion of remote consultations (10-percentage-point increase)	1.04 [1.01, 1.07]*	1.03 [1.00, 1.07]	1.02 [0.95, 1.09]	1.03 [0.99, 1.07]
Sex (cf. male)				
Female	0.80 [0.63, 1.02]	1.02 [0.80, 1.30]	0.59 [0.45, 0.76]**	0.99 [0.74, 1.32]
Ethnicity (cf. white)				
Black, Black British, Caribbean or African	1.12 [0.89, 1.42]	1.74 [1.37, 2.20]**	1.24 [0.74, 2.08]	2.23 [1.63, 3.04]**
Asian or Asian British	0.43 [0.21, 0.88]*	0.61 [0.31, 1.22]	0.33 [0.12, 0.90] *	0.79 [0.40, 1.56]
Mixed or multiple ethnic groups	0.89 [0.69, 1.14]	1.01 [0.71, 1.43]	1.20 [0.75, 1.93]	1.31 [0.85, 2.01]
Other ethnic group	0.56 [0.29, 1.08]	1.01 [0.38, 2.67]	0.63 [0.23, 1.76]	1.06 [0.33, 3.44]
Age	0.96 [0.96, 0.97]**	0.97 [0.96, 0.98]**	1.01 [0.99, 1.04]	0.97 [0.97, 0.98]**
IMD decile	0.87 [0.81, 0.93]**	0.91 [0.84, 0.99]*	1.17 [0.97, 1.42]	0.93 [0.84, 1.04]
History of anxiety	1.29 [1.07, 1.55]**	0.91 [0.72, 1.16]	0.87 [0.64, 1.19]	0.78 [0.58, 1.05]
History of depression	1.41 [1.07, 1.86]*	0.90 [0.74, 1.10]	0.68 [0.53, 0.87]**	0.71 [0.56, 0.92]**
History of SMI	17.47 [13.16, 23.21]**	45.18 [31.55, 64.70]**	43.84 [29.64, 64.85]**	72.38 [44.64, 117.36]**
Total GP consultations	1.07 [1.06, 1.09]**	1.05 [1.03, 1.08]**	1.03 [1.01, 1.06]*	1.02 [1.00, 1.05]

All associations were estimated using two-sided GEEs clustering by GP practice and adjusting for demographic and clinical characteristics, total number of consultations and subperiods.

95% CIs are reported in square brackets.

* and ** indicate significance at the 95% and 99% level, respectively.

Sensitivity analyses using complete-case data yielded estimates in the same direction but with slightly larger magnitudes (Supplementary Table 11). The association with psychiatric hospital admissions was statistically significant in the complete-case model (IRR 1.04, 95% CI 1.01–1.08, *P* = 0.022) but attenuated to nonsignificance after imputation (IRR 1.03, 95% CI 1.00–1.07, *P* = 0.076). This suggests that excluding patients with missing data inflated the risk in the complete-case analysis. Model stability metrics (QIC) were consistent across both approaches, confirming that these differences were driven by sample composition rather than model fit.

Female patients had shorter hospital stays than males (IRR 0.59, 95% CI 0.45–0.76, *P* < 0.001), although no differences were found for other outcomes. Compared with white patients, Asian or Asian British patients had fewer emergency contacts (IRR 0.43, 95% CI 0.21–0.88, *P* = 0.021) and shorter admissions (IRR 0.33, 95% CI 0.12–0.90, *P* = 0.032), whereas Black, Black British, Caribbean or African patients had higher rates of psychiatric hospital admissions (IRR 1.74, 95% CI 1.37–2.20, *P* < 0.001) and were more likely to experience MHA admissions (IRR 2.23, 95% CI 1.63–3.04, *P* < 0.001), highlighting ethnic disparities in service use. Increasing age and lower area deprivation were associated with reduced acute mental health service use across most outcomes.

History of SMI was strongly associated with all outcomes, with IRRs ranging from 17.5 to 72.4 (all *P* < 0.001). History of depression was linked to more emergency contacts (IRR 1.41, 95% CI 1.07–1.86, *P* = 0.015), but fewer inpatient bed-days (IRR 0.68, 95% CI 0.53–0.87, *P* = 0.002) and lower likelihood of MHA admissions (IRR 0.71, 95% CI 0.56–0.92, *P* = 0.009). History of anxiety was associated with more emergency contacts (IRR 1.29, 95% CI 1.07–1.55, *P* = 0.006) but was not significantly associated with other outcomes. Furthermore, there was no evidence of significant variation in acute mental health outcomes across the five study subperiods, despite the context changes due to the pandemic. Full estimates for period effects are provided in Supplementary Table 8.

Interaction analyses indicated that the observed effects of remote consultations were broadly consistent across demographic subgroups. The interaction between remote consultations and female sex for inpatient bed-days reached statistical significance (IRR 1.12, *P* = 0.019). No other interactions were found with age, ethnicity or IMD. Full interaction analyses are presented in Supplementary Tables 12 and 13.

## Discussion

### Key findings

In this large retrospective cohort study linking primary and secondary care data, we found that a higher proportion of remote consultations in primary care was associated with a modest increase in emergency contacts with mental health liaison teams. Specifically, for every 10-percentage-point rise in remote consultations, emergency presentations increased by 4%.

However, we found no evidence of an association between consultation modality and psychiatric hospital admissions, inpatient bed-days or compulsory detention under the MHA. While our sensitivity analysis of complete cases suggested a link with admissions, the effect disappeared once we corrected for missing data. These null findings suggest that, despite the increase in emergency contacts, remote consultations do not appear to result in severe clinical deterioration that would require increased hospitalization or involuntary detention. This provides reassurance that the shift toward remote primary care did not compromise patient safety regarding severe outcomes.

In addition, the use of acute mental health services differed based on demographic and clinical characteristics. The effect sizes observed for Black ethnicity and history of SMI highlight that the modality of primary care access is secondary to underlying clinical complexity and structural disparities in determining crisis outcomes.

### Interpretation and comparison with literature

The association between remote consultations and emergency contacts may be explained by several factors. First, as 96% of remote contacts in this study were telephone consultations, the lack of visual cues required for a comprehensive mental health evaluation may have prompted clinicians to direct patients to emergency departments as a precautionary measure. This is consistent with the findings of a systematic review of UK primary care that found that, while remote consulting is often considered convenient, face-to-face interactions are frequently preferred for complex care^25^. Second, the shift to remote care coincided with social lockdowns, which substantially disrupted support networks. In this context, a remote consultation often served as the only access point for patients in acute distress. Consequently, the rise in emergency presentations may reflect the collapse of wider community resources during the pandemic, leaving emergency departments as the only available option. Finally, reverse causation is plausible. Patients who are already deteriorating may be harder to engage face to face, or GPs may proactively call patients they know are at risk.

Studies conducted in other settings have reported contrasting findings. For example, a primary care-based telepsychiatry program for older adults was found to significantly reduce emergency visits and hospitalizations^26^. Similarly, other studies have reported reduced hospital use associated with telepsychiatry services^27,28^. However, these studies examined specialist services rather than GPs. This distinction suggests that, while specialist telepsychiatry may stabilize patients, the remote modality in primary care might function as a triage pathway to secondary care as a safety net.

Furthermore, our null findings challenge the concerns regarding the potential harms of remote consultations, as they suggest that using this modality does not necessarily translate into higher rates of psychiatric hospital admissions, longer hospital stays or compulsory detentions. This aligns with an evaluation of remote mental health services in South London, which found that, while digital exclusion remains a concern, remote care is an acceptable and effective alternative for service users when personal choice is accounted for^29^.

In terms of demographic and clinical characteristics, our findings confirm that these factors are strongly associated with acute outcomes. Consistent with previous studies from South London and other settings, we observed higher rates of compulsory psychiatric admissions among people from Black ethnic groups compared with white populations^30–32^. In addition, history of SMI was consistently one of the strongest predictors of acute mental health service use, including emergency visits and psychiatric hospitalizations^33–35^.

### Strengths and limitations

This study provides an important contribution to understanding the associations between consultation modality in primary care and acute mental health outcomes in secondary care. The study covers the period from January 2019 to December 2021, spanning both prepandemic and pandemic phases, which allows a longer-term perspective on the association of GP consultation modalities and mental health outcomes than many previous studies provide.

A key strength of this work is the robust methodological approach using GEE to account for patient clustering within GP practices and MICE to address missing data. The comparison between our primary (MICE) and sensitivity (complete-case) analyses highlighted the importance of handling missingness in routine data studies. Furthermore, the study benefits from a data linkage between two large and well-established databases, CRIS and LDN, enabling a detailed examination of relevant demographic confounders.

However, several limitations must be considered. First, this is an observational retrospective study, and the findings represent associations, not causal effects. As with all routine data analyses, we could not determine the specific reason for the GP consultation or the patient’s preference. It is possible that some patients sought care due to physical health needs, with secondary mental health concerns identified during the consultation, precipitating the contact with mental health liaison teams. In addition, reverse causation and time-varying confounding are possible because patients who are already deteriorating may be more likely to receive remote follow-up from their GP.

Second, while we adjusted for period effects to account for changes due to the COVID-19 pandemic, primary care capacity and hospital admission thresholds may have influenced these trends beyond statistical adjustment can control. For example, social distancing restrictions or fear of infection may have influenced help-seeking behaviors independent of consultation modality.

Third, our analysis was restricted to patients registered in Lambeth, a highly and densely populated borough in London. While this provides valuable data on health inequalities, results may differ in rural or less deprived areas where access to digital services and emergency care varies. Furthermore, data quality constraints meant that variables such as severity of mental illness, content of GP consultations, and outcomes occurring outside of SLaM were unavailable.

Finally, most remote consultations in this dataset were delivered via telephone. Therefore, our findings regarding the risks of remote assessment cannot be generalized to video-based care, which may have a different profile.

### Implications for future practice and research

For clinical practice, the association between remote consultations and increased emergency contacts with mental health liaison teams suggests that remote triage processes may need refinement. If remote contacts make it more difficult to identify escalating mental health symptoms, primary care providers may benefit from specific safety protocols. These could include reevaluating the threshold to offer a face-to-face assessment when a patient presents ambiguous distress, so that the emergency department does not become the default alternative. In addition, clinicians should remain aware that a request for a remote consultation from a patient with a history of SMI may indicate higher risk or difficulty engaging with standard care.

For future research, studies could compare telephone and video modalities to determine whether the presence of visual cues mitigates the risk of emergency presentations. Future research could also examine potential disparities in access to, uptake of and outcomes of remote GP consultations among different demographic groups, including those with different mental health conditions, physical and mental disabilities, lower digital literacy, limited access to technology and differing cultural beliefs around healthcare. Finally, qualitative work exploring patient and staff experiences of remote consultations can offer valuable insights into the acceptability, feasibility and satisfaction with telehealth options in mental health care to further understand this reality, particularly in line with the complexities of GP consultations service delivery.

## Conclusion

Greater use of remote consultations in primary care was associated with a modest increase in emergency contacts with mental health liaison teams but not with psychiatric hospital admissions, inpatient bed-days or compulsory detentions. These findings may reflect precautionary measures adopted by clinicians lacking visual cues due to the predominance of telephone contacts, or reverse causation where deteriorating patients are followed up remotely. However, the lack of associations with admissions or compulsory detentions provides reassurance that the shift to remote primary care did not compromise patient safety regarding severe outcomes. Future research is needed to determine whether video consultations optimize outcomes.

## Methods

### Ethical approval

This study complies with all relevant ethical regulations. We have received ethical approval from the South Central Oxford C Research Ethics Committee (REC) for use of the CRIS system as a secondary research database (REC reference: 23/SC/0257). This study received approval by CRIS (reference: 22-040) on 8 June 2022 (including external data linkages). Consent was not needed as we used a secondary research database.

### Study design

This is a retrospective cohort study using an exposure–outcome design investigating the relationship between primary care consultation modalities, categorized as face to face and remote (via telephone, text, email and video calls), and acute mental health service use. Specifically, it examines the number of emergency contacts with mental health liaison teams, psychiatric hospital admissions, inpatient bed-days and involuntary MHA admissions among patients with depression, anxiety and SMI (including bipolar and psychotic disorders) residing in a specific geographic catchment area, the London borough of Lambeth, before and during the COVID-19 pandemic. In this UK-based study, emergency contacts with mental health liaison teams refer to contacts in Accident and Emergency (A&E) departments of general hospitals that required intervention from mental health teams and were consequently recorded in the electronic health records of the mental health service.

### Data sources

This study utilized data from the CRIS system, which extracts predefined search parameters from the electronic patient records of the SLaM NHS Trust, a major mental health care provider covering a defined geographic catchment area across four London boroughs (Croydon, Lambeth, Lewisham and Southwark; approximately 1.3 million residents), which has deployed fully electronic health records across all services since 2006. CRIS provides researchers access to de-identified data from these records within a robust data governance and security framework^36,37^. Since the development of the platform in 2007–2008, CRIS data have been linked to a range of external data sources, including, as in this study, to primary care consultation data from the London borough of Lambeth, stored within the LDN^38^ via a bespoke trusted research environment administered by SLaM’s Clinical Data Linkage Service^36,37^. The linkage between LDN and CRIS has been previously used to investigate health inequalities and service use pathways^9,33^. Of relevance to this study, Lambeth represents around one quarter of SLaM’s catchment population and all of Lambeth’s catchment receives public mental health services from SLaM.

### Study population and case definition

The study cohort included patients registered with an LDN GP within the study period (1 January 2019 to 31 December 2021) who had a diagnosis of anxiety, depression or SMI, including bipolar and psychotic disorders, according to the Systematized Nomenclature of Medicine Clinical Terms (SNOMED), a structured clinical vocabulary used in electronic health records. Patients were excluded if they were under 18 years of age at exposure, were not registered to a GP or received zero consultations during the exposure period, or died before the start of the outcome period.

The study period was divided into five exposure–outcome subperiods, equally distributed over the full timeline (Fig. 1). Each exposure period was of 6 months in length. We calculated ‘active days’, defined as the number of days the patient was registered with a GP during this window. The exposure metric was the proportion of remote GP consultations experienced during that time, which was calculated as the number of remote GP consultations divided by the total number of GP consultations with valid (nonmissing) modalities. At the end of each exposure subperiod, the outcome measures were then calculated for each patient in the subsequent 6-month outcome period, adjusting for ‘days at risk’, which restricted the number of follow-up days if the patient died during the outcome phase.

The data from each exposure–outcome subperiod were then recombined. If a patient featured in more than one eligible subperiod, only one set of their data was used in the recombination, selected by random. The associated subperiod for each final patient was recorded for future use when adjusting for confounders, allowing mitigation of the effect of the pandemic peaks.

### Variables and outcome measures

The primary outcome measures were the number of: (1) emergency contacts with mental health liaison teams, (2) psychiatric hospital admissions, (3) inpatient bed-days and (4) involuntary admissions under the MHA, as recorded in mental healthcare (CRIS). Inpatient bed-days were calculated within each outcome period, and admission episodes extending beyond the end of the period were truncated. For the final outcome, we counted admissions that were classified as compulsory detention under the MHA at the time of admission.

In addition, the following data were extracted for each patient, using the most recent records at the time of extraction: date of death, age, sex, ethnicity, neighborhood socioeconomic deprivation (measured by the IMD), history of depression, anxiety or SMI (including bipolar and psychotic disorders) according to SNOMED, admission date, discharge date, GP consultation date and GP consultation modality.

### Missing data handling

Patterns of missing data were examined at patient and GP-practice levels. Missingness was associated with observed characteristics, including demographic and practice-level data recording practices, supporting a missing-at-random assumption. MICE was used to address incomplete data for sex, ethnicity, IMD and proportion of remote consultations, generating five imputed datasets that incorporated all analytical variables. Estimates from the imputed datasets were pooled using Rubin’s rules.

### Data analysis

The analyses were performed within a Jupyter Notebook using Python (version 3.11) with the statsmodels (version 0.14.0) and SciPy (version 1.16.2) packages.

Before modeling, the distribution of each outcome variable was examined. Normality was tested using D’Agostino’s *K*^2^ test, and dispersion was evaluated by comparing the variance to the mean. All outcomes demonstrated deviation from normality and high right skew with a high frequency of zeros. The variance exceeded the mean and showed overdispersion, which supported the use of negative binomial distribution.

Associations between the proportion of remote consultations and acute mental health service use were estimated using GEEs to account for clustering of patients within GP practices. Negative binomial GEEs were fitted for all four outcomes, including an offset for days at risk to adjust for variation in individual follow-up time. Complete-case analyses were conducted as a sensitivity check. Results are reported as IRRs with 95% CIs.

The proportion of remote consultations during the exposure window was analyzed as a continuous variable and scaled per 10-percentage-point increase. All models were adjusted for demographics (age, sex, ethnicity and IMD decile), clinical history (SMI, anxiety and depression), study subperiod and total GP consultations.

Interactions were tested between consultation modality and age, sex, ethnicity and IMD to examine whether the association between remote care and outcomes differed across demographic groups.

Model fit was assessed using complementary approaches. The QIC was used to compare model structures and optimize the negative binomial dispersion parameter (alpha) for each outcome. Missing data were addressed using MICE, and the impact of imputation on model uncertainty was evaluated using FMI and RIV metrics. Finally, model assumptions were examined using DHARMa-derived QQ plots and residuals-versus-predicted value plots.

All statistical tests were two-sided, and *P* values <0.05 were considered statistically significant.

### Information governance

Data are stored in a third-party database, the Maudsley Biomedical Research Centre CRIS database, which provides access to pseudonymised data derived from source electronic medical records. These data can only be accessed by authorized individuals from within a secure firewall (that is, the data cannot be sent elsewhere).

### Reporting summary

Further information on research design is available in the Nature Portfolio Reporting Summary linked to this article.

## Supplementary information

Supplementary Information Supplementary Tables 1–13 and Figs. 1–3.

Reporting Summary

## Data Availability

The data that support the findings of this study are pseudonymized electronic health records from the SLaM NHS Foundation Trust (CRIS) and LDN. Due to ethical and information governance restrictions regarding patient confidentiality, these data are not publicly available. Access to the data is restricted to researchers with appropriate honorary contracts and approvals. Information regarding the CRIS system and the formal application procedures required for external researchers can be found at https://www.maudsleybrc.nihr.ac.uk/facilities/clinical-record-interactive-search-cris/.

## Code availability

Analytic scripts used to generate the final results presented in this article, including data cleaning and analysis (avantgarda/gp-consultation-modality-mental-health: v1.0.0), are available via Zenodo at 10.5281/zenodo.17949821 (ref. ^39^).
