# Supplementary material for: Primary care consultation modality and acute mental health service use in adults
Source: Nat Ment Health. 2026 Mar 17;4(4):574–81. doi: 10.1038/s44220-026-00605-9 (PMC13076198; doi:10.1038/s44220-026-00605-9)
Supplement: Supplementary file 1 — Supplementary Tables 1–13 and Figs. 1–3. [file 44220_2026_605_MOESM1_ESM.pdf]

nature mental health

Supplementary information https://doi.org/10.1038/s44220-026-00605-9

Primary care consultation modality and acute mental health service use in adults

In the format provided by the authors and unedited

Supplementary Materials

Contents

Section A: Missingness and Imputation 2

Supplementary Table 1: Missing data counts and proportions 2

Supplementary Table 2: Associations between missingness and observed characteristics 3

Supplementary Figure 1: Variation in missingness rates across GP practices 6

Supplementary Table 3: Variables included in the Multiple Imputation by Chained Equations (MICE) model 7

Supplementary Table 4: Comparison of observed and post-imputation data distributions for categorical variables 8

Supplementary Table 5: Comparison of observed and post-imputation data distributions for continuous variables 9

Section B: Sensitivity Analysis Population 10

Supplementary Table 6: Baseline characteristics of the complete-case cohort 10

Section C: Outcome Distributions 11

Supplementary Table 7: Descriptive statistics for outcome distributions 11

Section D: Regression Results and Model Diagnostics 12

Supplementary Table 8: MICE (primary) results 12

Supplementary Table 9: Goodness-of-fit statistics (QIC, Optimised Dispersion Parameters) 14

Supplementary Table 10: Imputation Diagnostics for the Primary Exposure (proportion of remote consultations) 15

Supplementary Figure 2: DHARMa-derived QQ plots for all outcomes (MICE and Complete case) 16

Supplementary Figure 3: Residuals versus predicted values plots for all outcomes (Complete case and MICE) 17

Supplementary Table 11: Complete-case (sensitivity) results 18

Section E: Interactions 20

Supplementary Table 12: Interaction analyses between consultation modality and demographic characteristics for the primary analysis (MICE) 20

Supplementary Table 13: Interaction analyses between consultation modality and demographic characteristics for complete case analysis 21

Section A: Missingness and Imputation

Supplementary Table 1: Missing data counts and proportions

Variables	Missing data (N=107,993)	
	n	%
Ethnicity	6819	6.31
Index of Multiple Deprivation	3927	3.64
Consultation modality	1380	1.28
Sex	8	0.01

Supplementary Table 2: Associations between missingness and observed characteristics

Observed characteristic	Sex			Ethnicity			IMD			Proportion of remote consultations		
	Complete group	Missing group	SMD	Complete group	Missing group	SMD	Complete group	Missing group	SMD	Complete group	Missing group	SMD
Age (median [IQR])	42.00 [30.50-58.00]	29.50 [27.00-35.62]	1.143	42.49 [31.00-58.00]	36.00 [27.50-52.00]	0.321	42.00 [30.50-58.00]	36.50 [28.50-51.50]	0.264	42.00 [30.50-58.00]	38.50 [30.00-53.50]	0.162
Sex n (%)												
Male	NA	NA	NA	40477 (40.0%)	2986 (43.8%)	0.077	41686 (40.1%)	1777 (45.3%)	0.105	42750 (40.1%)	713 (51.7%)	0.234
Female	NA	NA	NA	60690 (60.0%)	3832 (56.2%)	0.077	62372 (59.9%)	2150 (54.7%)	0.105	63855 (59.9%)	667 (48.3%)	0.234
Ethnicity n (%)												
White	46182 (45.6%)	5 (71.4%)	0.542	NA	NA	NA	44427 (45.6%)	1760 (47.3%)	0.034	45595 (45.6%)	592 (45.9%)	0.004
Black, Black British, Caribbean or African	21821 (21.6%)	0 (0.0%)	0.742	NA	NA	NA	21324 (21.9%)	497 (13.3%)	0.225	21591 (21.6%)	230 (17.8%)	0.096
Asian or Asian British	7380 (7.3%)	0 (0.0%)	0.397	NA	NA	NA	6920 (7.1%)	460 (12.4%)	0.178	7295 (7.3%)	85 (6.6%)	0.028
Mixed or multiple ethnic groups	23026 (22.8%)	2 (28.6%)	0.133	NA	NA	NA	22222 (22.8%)	806 (21.6%)	0.028	22674 (22.7%)	354 (27.4%)	0.109
Other ethnic group	2758 (2.7%)	0 (0.0%)	0.237	NA	NA	NA	2558 (2.6%)	200 (5.4%)	0.141	2728 (2.7%)	30 (2.3%)	0.026
IMD decile n (%)												
1 – most deprived	164 (0.2%)	0 (0.0%)	0.056	160 (0.2%)	4 (0.1%)	0.031	NA	NA	NA	162 (0.2%)	2 (0.1%)	0.002
2	20429 (19.6%)	2 (25.0%)	0.129	19098 (19.6%)	1333 (20.2%)	0.014	NA	NA	NA	20127 (19.6%)	304 (22.8%)	0.078
3	26155 (25.1%)	3 (37.5%)	0.269	24429 (25.1%)	1729 (26.1%)	0.025	NA	NA	NA	25828 (25.1%)	330 (24.7%)	0.010
4	22754 (21.9%)	1 (12.5%)	0.250	21384 (21.9%)	1371 (20.7%)	0.030	NA	NA	NA	22491 (21.9%)	264 (19.8%)	0.052
5	14019 (13.5%)	1 (12.5%)	0.029	13125 (13.5%)	895 (13.5%)	0.002	NA	NA	NA	13836 (13.5%)	184 (13.8%)	0.009
6	12218 (11.7%)	1 (12.5%)	0.023	11430 (11.7%)	789 (11.9%)	0.006	NA	NA	NA	12064 (11.7%)	155 (11.6%)	0.004

7	4076 (3.9%)	0 (0.0%)	0.286	3809 (3.9%)	267 (4.0%)	0.007	NA	NA	NA	4041 (3.9%)	35 (2.6%)	0.074
8	2785 (2.7%)	0 (0.0%)	0.235	2632 (2.7%)	153 (2.3%)	0.025	NA	NA	NA	2746 (2.7%)	39 (2.9%)	0.015
9	1158 (1.1%)	0 (0.0%)	0.150	1096 (1.1%)	62 (0.9%)	0.019	NA	NA	NA	1141 (1.1%)	17 (1.3%)	0.015
10 – least deprived	300 (0.3%)	0 (0.0%)	0.076	288 (0.3%)	12 (0.2%)	0.023	NA	NA	NA	295 (0.3%)	5 (0.4%)	0.015
Clinical history n (%)												
History of anxiety	54403 (50.4%)	6 (75.0%)	0.526	50699 (50.1%)	3710 (54.4%)	0.086	52721 (50.7%)	1688 (43.0%)	0.154	53782 (50.4%)	627 (45.4%)	0.100
History of depression	86469 (80.1%)	5 (62.5%)	0.396	81429 (80.5%)	5045 (74.0%)	0.155	83133 (79.9%)	3341 (85.1%)	0.137	85367 (80.1%)	1107 (80.2%)	0.004
History of SMI	5889 (5.5%)	0 (0.0%)	0.340	5534 (5.5%)	355 (5.2%)	0.012	5713 (5.5%)	176 (4.5%)	0.046	5804 (5.4%)	85 (6.2%)	0.031
Study sub-period n (%)												
1	24507 (22.7%)	1 (12.5%)	0.270	23001 (22.7%)	1507 (22.1%)	0.015	23532 (22.6%)	976 (24.9%)	0.053	24171 (22.7%)	337 (24.4%)	0.041
2	22609 (20.9%)	1 (12.5%)	0.228	21194 (20.9%)	1416 (20.8%)	0.004	21784 (20.9%)	826 (21.0%)	0.002	22270 (20.9%)	340 (24.6%)	0.090
3	19042 (17.6%)	1 (12.5%)	0.144	17898 (17.7%)	1145 (16.8%)	0.024	18356 (17.6%)	687 (17.5%)	0.004	18806 (17.6%)	237 (17.2%)	0.012
4	19331 (17.9%)	2 (25.0%)	0.174	18105 (17.9%)	1228 (18.0%)	0.003	18646 (17.9%)	687 (17.5%)	0.011	19099 (17.9%)	234 (17.0%)	0.025
5	22496 (20.8%)	3 (37.5%)	0.373	20976 (20.7%)	1523 (22.3%)	0.039	21748 (20.9%)	751 (19.1%)	0.044	22267 (20.9%)	232 (16.8%)	0.104
Death at follow-up n (%)	1138 (1.1%)	0 (0.0%)	0.146	1089 (1.1%)	49 (0.7%)	0.038	1118 (1.1%)	20 (0.5%)	0.064	1131 (1.1%)	7 (0.5%)	0.063
Service use and outcomes (median [IQR])												
Total GP consultation rate	0.02 [0.01-0.03]	0.03 [0.01-0.04]	0.124	0.02 [0.01-0.03]	0.02 [0.01-0.03]	0.044	0.02 [0.01-0.03]	0.02 [0.01-0.03]	0.005	0.02 [0.01-0.03]	0.01 [0.01-0.01]	0.651
Proportion of remote consultations	0.50 [0.00-0.67]	0.50 [0.30-0.81]	0.243	0.50 [0.00-0.67]	0.50 [0.00-0.67]	0.009	0.50 [0.00-0.67]	0.50 [0.00-0.67]	0.000	NA	NA	NA
Rate of consultations with missing modality	0.00 [0.00-0.00]	0.00 [0.00-0.00]	0.014	0.00 [0.00-0.00]	0.00 [0.00-0.00]	0.108	0.00 [0.00-0.00]	0.00 [0.00-0.00]	0.048	0.00 [0.00-0.00]	0.01 [0.01-0.01]	0.809

Emergency contact with mental health liaison teams rate	0.00 [0.00-0.00]	0.00 [0.00-0.00]	0.080	0.00 [0.00-0.00]	0.00 [0.00-0.00]	0.005	0.00 [0.00-0.00]	0.00 [0.00-0.00]	0.017	0.00 [0.00-0.00]	0.00 [0.00-0.00]	0.005
Psychiatric hospital admission rate	0.00 [0.00-0.00]	0.00 [0.00-0.00]	0.073	0.00 [0.00-0.00]	0.00 [0.00-0.00]	0.002	0.00 [0.00-0.00]	0.00 [0.00-0.00]	0.028	0.00 [0.00-0.00]	0.00 [0.00-0.00]	0.032
Inpatient bed-days rate	0.00 [0.00-0.00]	0.00 [0.00-0.00]	0.067	0.00 [0.00-0.00]	0.00 [0.00-0.00]	0.015	0.00 [0.00-0.00]	0.00 [0.00-0.00]	0.013	0.00 [0.00-0.00]	0.00 [0.00-0.00]	0.069
MHA admission rate	0.00 [0.00-0.00]	0.00 [0.00-0.00]	0.061	0.00 [0.00-0.00]	0.00 [0.00-0.00]	0.009	0.00 [0.00-0.00]	0.00 [0.00-0.00]	0.032	0.00 [0.00-0.00]	0.00 [0.00-0.00]	0.033

IMD: Index of Multiple Deprivation; IQR: Interquartile Range; MHA: Mental Health Act; NA: Not Applicable; SMD: Standardised Mean Difference; SMI: Severe Mental Illness.
Rates are calculated per days at risk. The proportion of remote consultations is calculated as the ratio of remote to total consultations per patient (range 0–1).
An absolute SMD > 0.1 indicates a meaningful imbalance between patients with complete vs. missing data.

Supplementary Figure 1: Variation in missingness rates across GP practices

The figure displays the percentage of missing data for four key variables across all included GP practices: ethnicity, Index of Multiple Deprivation (IMD), sex, and consultation modality. Each horizontal bar represents an individual GP practice. The vertical dashed line indicates the mean percentage of missing data across all practices. Red bars indicate practices where missing data rates exceeded the mean by more than one standard deviation (> Mean + 1 SD), demonstrating significant practice-level variation in data recording.

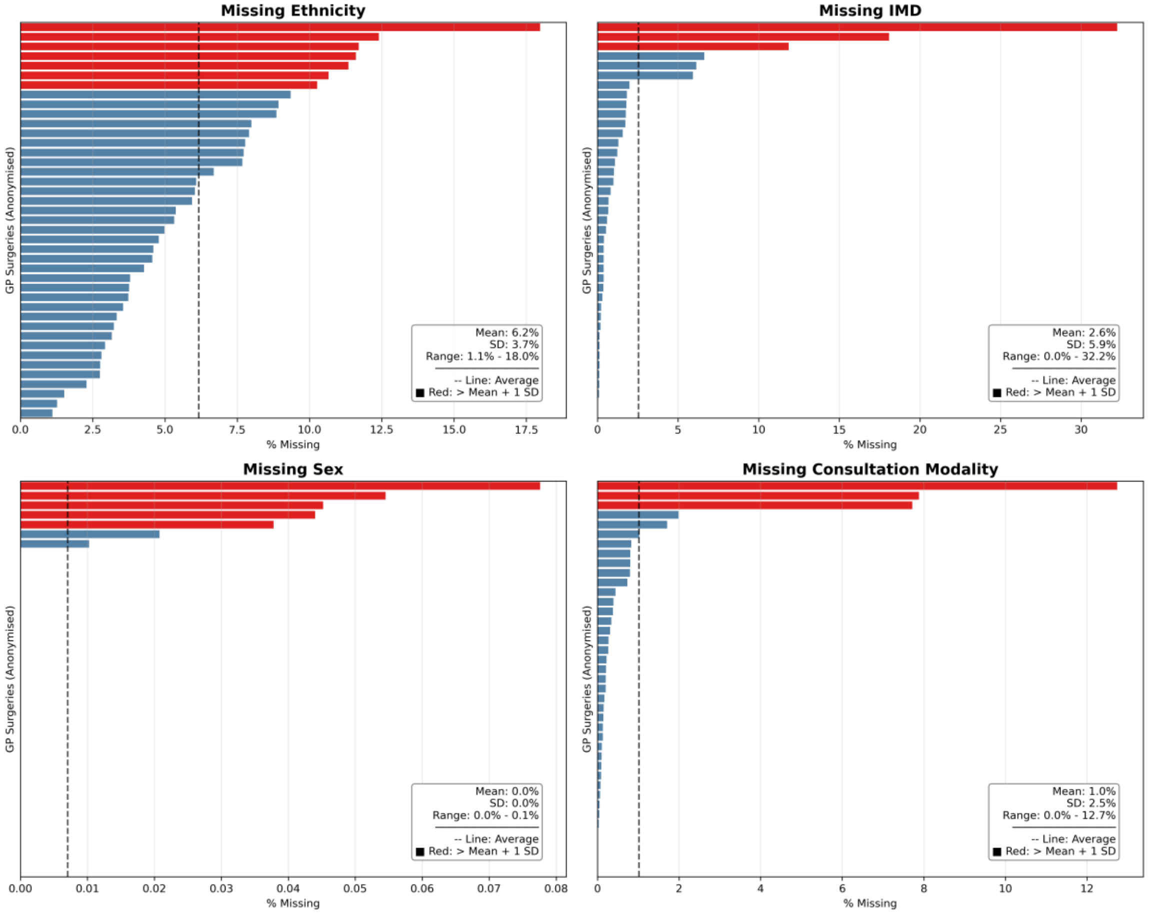

Supplementary Table 3: Variables included in the Multiple Imputation by Chained Equations (MICE) model

Variable category	Variable name	Role in imputation
Demographics	Age	Predictor
	Sex	Imputed
	Ethnicity	Imputed
	IMD	Imputed
Exposure	Proportion of remote consultations	Imputed
	Total GP consultations (count and rate per active days)	Predictor
	Remote consultations (count and rate per active days)	Predictor
	Face-to-face consultations (count and rate per active days)	Predictor
	Consultations with missing modality (count and rate per active days)	Predictor
Clinical history	History of anxiety	Predictor
	History of depression	Predictor
	History of SMI	Predictor
Acute mental health service use outcomes	Emergency contacts with mental health liaison teams (count and rate per days at risk)	Predictor
	Psychiatric hospital admissions (count and rate per days at risk)	Predictor
	Inpatient bed-days (count and rate per days at risk)	Predictor
	MHA admissions (count and rate per days at risk)	Predictor
Follow-up metrics	Active days in exposure period	Predictor
	Days at risk	Predictor
	Death at follow-up	Predictor

Supplementary Table 4: Comparison of observed and post-imputation data distributions for categorical variables

Variable	Level	Observed (Complete cases)	Post-imputation (Total)	Imputed count N
Sex	Male	43463	43465	2
	Female	64522	64528	6
Ethnicity	White	46187	49309	3122
	Black, Black British, Caribbean or African	21821	23171	1350
	Asian or Asian British	7380	7892	512
	Mixed or multiple ethnic groups	23028	24641	1613
	Other ethnic group	2758	2980	222
IMD decile	1 (most deprived)	164	167	3
	2	20431	21141	710
	3	26158	27194	1036
	4	22755	23683	928
	5	14020	14536	516
	6	12219	12639	420
	7	4076	4218	142
	8	2785	2893	108
	9	1158	1204	46
	10 (least deprived)	300	318	18

Supplementary Table 5: Comparison of observed and post-imputation data distributions for continuous variables

Variable	Dataset	N	Mean (SD)	Median [IQR]	Min - Max
Proportion of remote consultations	Observed	106613	0.43 (0.36)	0.50 [0.00–0.67]	0.00 – 1.00
	Post-imputation	107993	0.42 (0.36)	0.50 [0.00–0.67]	0.00 – 1.00

Section B: Sensitivity Analysis Population

Supplementary Table 6: Baseline characteristics of the complete-case cohort

Variables<br>N	Overall<br>106613	Sub-period 1<br>24171	Sub-period 2<br>22270	Sub-period 3<br>18806	Sub-period 4<br>19099	Sub-period 5<br>22267
Age (years), mean (SD)	45.7 (18.0)	45.8 (18.2)	46.2 (18.1)	46.4 (17.8)	45.7 (17.9)	44.7 (17.8)
Sex n (%)						
Male	42750 (40.1)	9793 (40.5)	9112 (40.9)	7507 (39.9)	7470 (39.1)	8868 (39.8)
Female	63855 (59.9)	14377 (59.5)	13157 (59.1)	11298 (60.1)	11627 (60.9)	13396 (60.2)
Ethnicity n (%)						
White	45595 (45.6)	10455 (46.1)	9490 (45.4)	7959 (45.0)	7994 (44.7)	9697 (46.7)
Black, Black British, Caribbean or African	21591 (21.6)	4635 (20.4)	4569 (21.9)	3971 (22.5)	4005 (22.4)	4411 (21.3)
Asian or Asian British	7295 (7.3)	1693 (7.5)	1510 (7.2)	1300 (7.4)	1325 (7.4)	1467 (7.1)
Mixed or multiple ethnic groups	22674 (22.7)	5337 (23.5)	4748 (22.7)	3927 (22.2)	4066 (22.7)	4596 (22.1)
Other ethnic groups	2728 (2.7)	564 (2.5)	571 (2.7)	516 (2.9)	492 (2.8)	585 (2.8)
IMD decile, median [Q1, Q3]	4.0 [3.0,5.0]	4.0 [3.0,5.0]	4.0 [3.0,5.0]	4.0 [3.0,5.0]	4.0 [3.0,5.0]	4.0 [3.0,5.0]
History of anxiety, n (%)	53782 (50.4)	11739 (48.6)	10955 (49.2)	9533 (50.7)	9747 (51.0)	11808 (53.0)
History of depression, n (%)	85367 (80.1)	19607 (81.1)	18008 (80.9)	15088 (80.2)	15290 (80.1)	17374 (78.0)
History of SMI, n (%)	5804 (5.4)	1278 (5.3)	1180 (5.3)	1058 (5.6)	1028 (5.4)	1260 (5.7)
Total consultations, median [Q1, Q3]	3.0 [1.0,5.0]	2.0 [1.0,5.0]	2.0 [1.0,5.0]	2.0 [1.0,5.0]	3.0 [2.0,6.0]	3.0 [2.0,6.0]
Proportion of remote consultations (continuous), median [Q1, Q3]	0.5 [0.0,0.7]	0.0 [0.0,0.5]	0.0 [0.0,0.5]	0.5 [0.0,0.8]	0.6 [0.5,0.9]	0.6 [0.5,0.8]

Section C: Outcome Distributions

Supplementary Table 7: Descriptive statistics for outcome distributions

Variable	Mean	Variance	Dispersion Ratio	Skew	Normality (p value)
Emergency contacts	0.020	0.090	5.230	31.770	< 0.001
Psychiatric hospital admissions	0.000	0.000	1.320	23.230	< 0.001
Inpatient bed-days	0.240	26.940	110.330	27.780	< 0.001
MHA admissions	0.000	0.000	1.220	26.140	< 0.001

MHA: Mental Health Act.

The Dispersion Ratio (Variance/Mean) indicates overdispersion (values significantly greater than 1), justifying the use of the Negative Binomial distribution in the analysis.

Normality was assessed using D'Agostino's K^2 test. The resulting p-values (<0.001) confirm that all outcome distributions deviate significantly from normality.

Section D: Regression Results and Model Diagnostics

Supplementary Table 8: MICE (primary) results

Covariate	N of emergency contacts with mental health liaison teams			N of psychiatric hospital admissions			N of inpatient bed-days			N of MHA admissions		
	Beta (SE)	IRR [95% CI]	P value	Beta (SE)	IRR [95% CI]	P value	Beta (SE)	IRR [95% CI]	P value	Beta (SE)	IRR [95% CI]	P value
Intercept	-8.68 (0.29)	0.00 [0.00, 0.00]	0.000	-10.93 (0.36)	0.00 [0.00, 0.00]	0.000	-8.80 (0.54)	0.00 [0.00, 0.00]	0.000	-12.00 (0.44)	0.00 [0.00, 0.00]	0.000
Proportion of remote consultations (10-percentage-point increase)	0.03 (0.02)	1.04 [1.01, 1.07]	0.021	0.03 (0.02)	1.03 [1.00, 1.07]	0.076	0.02 (0.03)	1.02 [0.95, 1.09]	0.573	0.03 (0.02)	1.03 [0.99, 1.07]	0.179
Sex (c.f. Male)												
Female	-0.22 (0.12)	0.80 [0.63, 1.02]	0.071	0.02 (0.13)	1.02 [0.80, 1.30]	0.890	-0.54 (0.13)	0.59 [0.45, 0.76]	0.000	-0.01 (0.15)	0.99 [0.74, 1.32]	0.922
Ethnicity (c.f. White)												
Black, Black British, Caribbean or African	0.12 (0.12)	1.12 [0.89, 1.42]	0.331	0.55 (0.12)	1.74 [1.37, 2.20]	0.000	0.22 (0.26)	1.24 [0.74, 2.08]	0.411	0.80 (0.16)	2.23 [1.63, 3.04]	0.000
Asian or Asian British	-0.85 (0.37)	0.43 [0.21, 0.88]	0.021	-0.49 (0.35)	0.61 [0.31, 1.22]	0.165	-1.11 (0.51)	0.33 [0.12, 0.90]	0.032	-0.24 (0.35)	0.79 [0.40, 1.56]	0.497
Mixed or multiple ethnic groups	-0.12 (0.13)	0.89 [0.69, 1.14]	0.354	0.01 (0.18)	1.01 [0.71, 1.43]	0.957	0.18 (0.24)	1.20 [0.75, 1.93]	0.452	0.27 (0.22)	1.31 [0.85, 2.01]	0.222
Other ethnic group	-0.57 (0.33)	0.56 [0.29, 1.08]	0.086	0.01 (0.50)	1.01 [0.38, 2.67]	0.986	-0.46 (0.52)	0.63 [0.23, 1.76]	0.381	0.06 (0.60)	1.06 [0.33, 3.44]	0.926
Age	-0.04 (0.00)	0.96 [0.96, 0.97]	0.000	-0.03 (0.00)	0.97 [0.96, 0.98]	0.000	0.01 (0.01)	1.01 [0.99, 1.04]	0.284	-0.03 (0.00)	0.97 [0.97, 0.98]	0.000
IMD decile	-0.14 (0.04)	0.87 [0.81, 0.93]	0.000	-0.10 (0.04)	0.91 [0.84, 0.99]	0.025	0.16 (0.10)	1.17 [0.97, 1.42]	0.104	-0.07 (0.05)	0.93 [0.84, 1.04]	0.201

Study sub-period (c.f. Sub-period 1)												
Sub-period 2	0.13 (0.15)	1.14 [0.85, 1.52]	0.391	0.11 (0.22)	1.12 [0.72, 1.72]	0.622	-0.35 (0.25)	0.71 [0.44, 1.14]	0.158	0.26 (0.25)	1.30 [0.79, 2.13]	0.296
Sub-period 3	0.24 (0.15)	1.27 [0.95, 1.71]	0.112	0.11 (0.19)	1.11 [0.78, 1.60]	0.559	-0.18 (0.20)	0.83 [0.57, 1.22]	0.348	0.26 (0.25)	1.30 [0.79, 2.13]	0.307
Sub-period 4	0.20 (0.18)	1.22 [0.87, 1.73]	0.250	0.15 (0.18)	1.16 [0.82, 1.64]	0.404	0.31 (0.20)	1.37 [0.92, 2.04]	0.126	0.35 (0.21)	1.41 [0.94, 2.12]	0.093
Sub-period 5	0.23 (0.18)	1.26 [0.89, 1.78]	0.195	0.14 (0.17)	1.16 [0.84, 1.60]	0.382	0.10 (0.23)	1.11 [0.71, 1.73]	0.655	0.31 (0.20)	1.36 [0.91, 2.02]	0.135
History of anxiety	0.25 (0.09)	1.29 [1.07, 1.55]	0.006	-0.09 (0.12)	0.91 [0.72, 1.16]	0.466	-0.14 (0.16)	0.87 [0.64, 1.19]	0.380	-0.25 (0.15)	0.78 [0.58, 1.05]	0.101
History of depression	0.34 (0.14)	1.41 [1.07, 1.86]	0.015	-0.10 (0.10)	0.90 [0.74, 1.10]	0.309	-0.39 (0.12)	0.68 [0.53, 0.87]	0.002	-0.34 (0.13)	0.71 [0.56, 0.92]	0.009
History of SMI	2.86 (0.15)	17.47 [13.16, 23.21]	0.000	3.81 (0.18)	45.18 [31.55, 64.70]	0.000	3.78 (0.20)	43.84 [29.64, 64.85]	0.000	4.28 (0.25)	72.38 [44.64, 117.36]	0.000
Total GP consultations	0.07 (0.01)	1.07 [1.06, 1.09]	0.000	0.05 (0.01)	1.05 [1.03, 1.08]	0.000	0.03 (0.01)	1.03 [1.01, 1.06]	0.020	0.02 (0.01)	1.02 [1.00, 1.05]	0.092

CI: Confidence Interval; IMD: Index of Multiple Deprivation; IRR: Incidence Rate Ratio; MHA: Mental Health Act; SE: Standard Error; SMI: Severe Mental Illness.
All associations were estimated using Generalised Estimating Equations clustering by GP practice. Models included an offset for time at risk to account for varying follow-up durations.

Supplementary Table 9: Goodness-of-fit statistics (QIC, Optimised Dispersion Parameters)

Outcome	Model specification	N	Optimised Dispersion Parameter (Alpha)	Goodness-of-fit (QIC)
Emergency contacts with mental health liaison teams	Complete case	96195	0.100	13770.87
	MICE (Pooled)	107993	0.500	15447.06
Psychiatric hospital admissions	Complete case	96195	3.000	2154.93
	MICE (Pooled)	107993	3.000	2450.75
Inpatient bed-days	Complete case	96195	0.500	108747.87
	MICE (Pooled)	107993	0.500	141173.88
MHA admissions	Complete case	96195	3.000	1447.43
	MICE (Pooled)	107993	3.000	1662.71

MHA: Mental Health Act; MICE: Multiple Imputation by Chained Equations; QIC: Quasi-likelihood under the Independence model Criterion

Alpha: Negative binomial dispersion parameter. The parameter was optimised for each outcome by selecting the value that minimised the QIC on a grid of candidate values.

Supplementary Table 10: Imputation Diagnostics for the Primary Exposure (proportion of remote consultations)

Outcome model	Fraction of Missing Information (FMI)	Relative Increase in Variance (RIV)
Emergency contacts with mental health liaison teams	0.667	0.001
Psychiatric hospital admissions	0.667	0.005
Inpatient bed-days	0.668	0.014
MHA admissions	0.667	0.006

Diagnostics are reported for the primary exposure: Proportion of remote consultations (scaled per 10-percentage-point increase).

Supplementary Figure 2: DHARMa-derived QQ plots for all outcomes (MICE and Complete case)

Panel A: Residual diagnostics for the primary analysis (Pooled MICE cohort). Panel B: Residual diagnostics for the sensitivity analysis (Complete Case cohort). Plots display the observed versus theoretical quantiles of standardised residuals simulated from the fitted Negative Binomial GEE models using the DHARMa package. KS: Kolmogorov-Smirnov test for uniformity.

Panel A. Residual diagnostics for the primary analysis (Pooled MICE cohort)

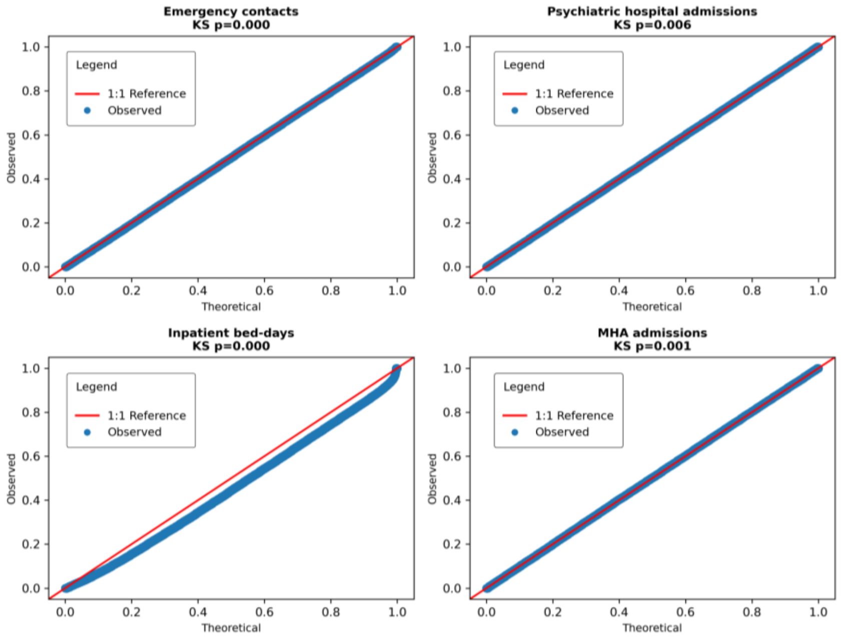

Panel B. Residual diagnostics for the sensitivity analysis (Complete Case cohort)

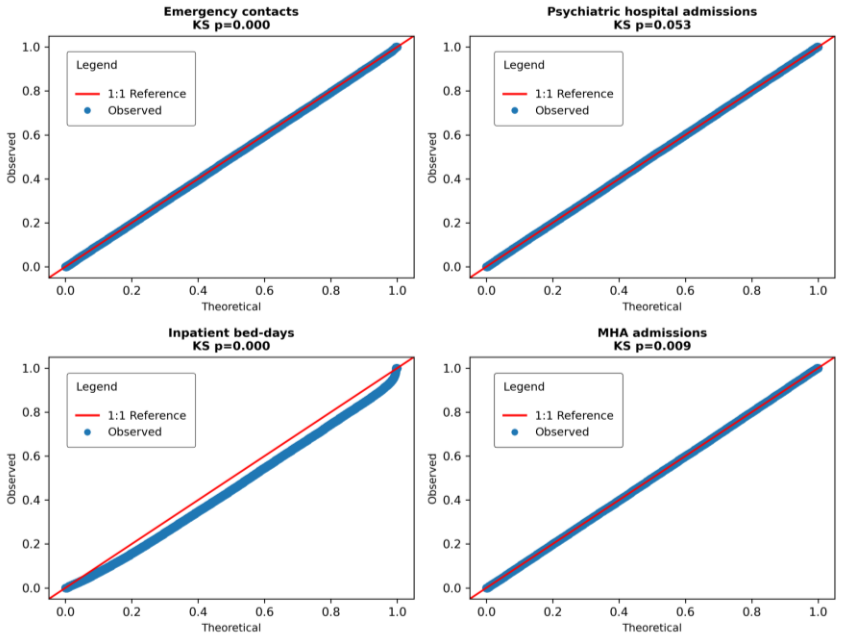

Supplementary Figure 3: Residuals versus predicted values plots for all outcomes (MICE and Complete Case)

Panel A: Diagnostic plots for the primary analysis (Pooled MICE cohort). Panel B: Diagnostic plots for the sensitivity analysis (Complete Case cohort). The scatter plots display standardised residuals simulated using the DHARMa package (y-axis) against the log-transformed predicted values from the Negative Binomial GEE models (x-axis).

Panel A. Diagnostic plots for the primary analysis (Pooled MICE cohort)

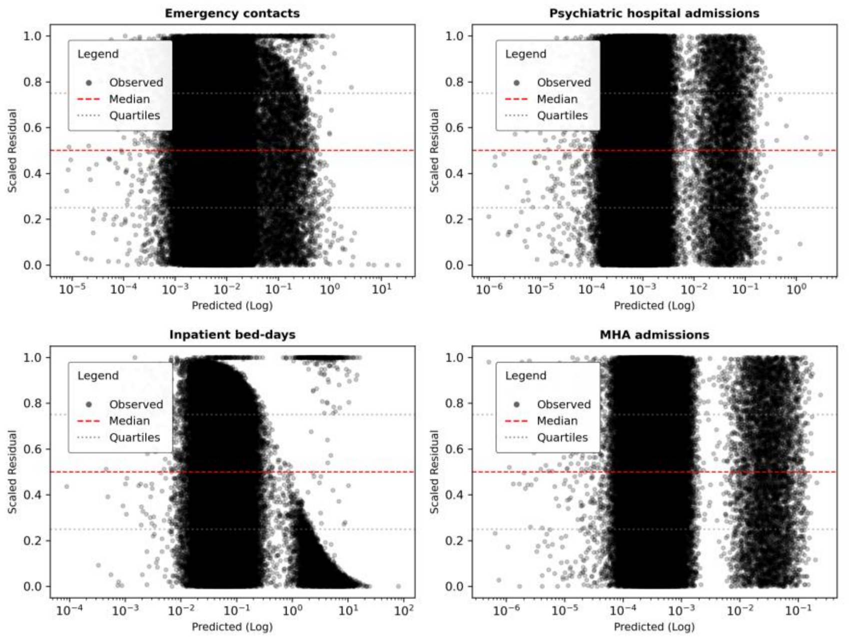

Panel B. Diagnostic plots for the sensitivity analysis (Complete Case cohort)

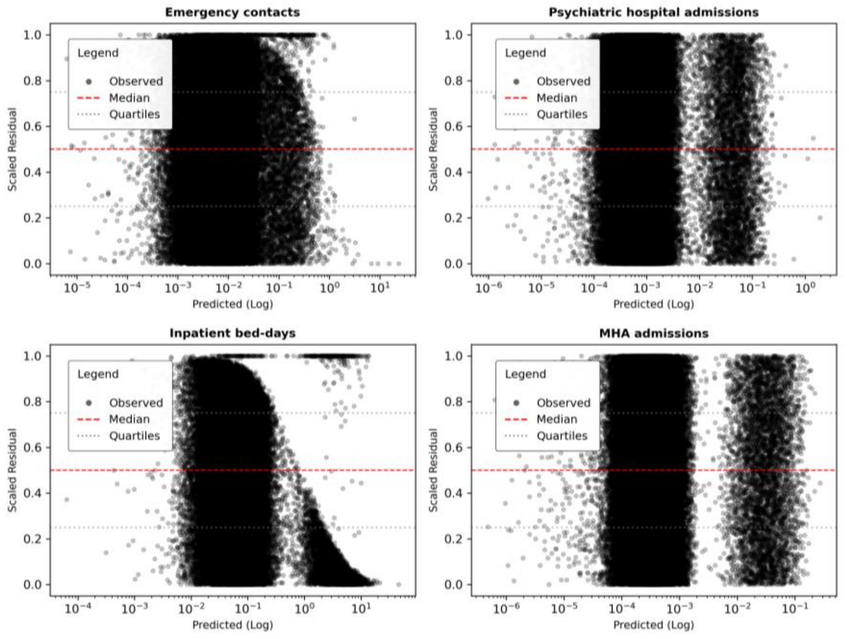

Supplementary Table 11: Complete-case (sensitivity) results

Covariate	N of emergency contacts with mental health liaison teams			N of psychiatric hospital admissions			N of inpatient bed-days			N of MHA admissions		
	Beta (SE)	IRR [95% CI]	P value	Beta (SE)	IRR [95% CI]	P value	Beta (SE)	IRR [95% CI]	P value	Beta (SE)	IRR [95% CI]	P value
Intercept	-8.50 (0.28)	0.00 [0.00, 0.00]	0.000	-10.88 (0.38)	0.00 [0.00, 0.00]	0.000	-8.68 (0.48)	0.00 [0.00, 0.00]	0.000	-12.01 (0.48)	0.00 [0.00, 0.00]	0.000
Proportion of remote consultations (10-percentage-point increase)	0.04 (0.02)	1.04 [1.00, 1.07]	0.034	0.04 (0.02)	1.04 [1.01, 1.08]	0.022	0.03 (0.03)	1.03 [0.97, 1.10]	0.305	0.04 (0.02)	1.04 [1.00, 1.08]	0.054
Sex (c.f. Male)												
Female	-0.28 (0.13)	0.76 [0.58, 0.98]	0.038	-0.03 (0.14)	0.97 [0.74, 1.27]	0.829	-0.53 (0.14)	0.59 [0.44, 0.78]	0.000	-0.02 (0.16)	0.98 [0.72, 1.35]	0.916
Ethnicity (c.f. White)												
Black, Black British, Caribbean or African	0.03 (0.12)	1.03 [0.82, 1.30]	0.792	0.52 (0.12)	1.68 [1.34, 2.11]	0.000	0.23 (0.26)	1.26 [0.76, 2.09]	0.370	0.76 (0.17)	2.15 [1.54, 2.99]	0.000
Asian or Asian British	-1.07 (0.34)	0.34 [0.18, 0.67]	0.002	-0.63 (0.36)	0.53 [0.26, 1.07]	0.078	-1.12 (0.46)	0.33 [0.13, 0.81]	0.015	-0.28 (0.41)	0.75 [0.34, 1.69]	0.491
Mixed or multiple ethnic groups	-0.17 (0.12)	0.84 [0.67, 1.07]	0.154	0.03 (0.17)	1.03 [0.75, 1.43]	0.839	0.27 (0.27)	1.30 [0.77, 2.19]	0.320	0.29 (0.22)	1.34 [0.87, 2.05]	0.187
Other ethnic group	-0.65 (0.32)	0.52 [0.28, 0.98]	0.044	-0.03 (0.42)	0.97 [0.42, 2.22]	0.941	-0.49 (0.47)	0.62 [0.24, 1.56]	0.306	0.02 (0.61)	1.02 [0.31, 3.35]	0.972
Age	-0.01 (0.00)	0.96 [0.95, 0.97]	0.000	-0.03 (0.00)	0.97 [0.96, 0.98]	0.000	0.01 (0.01)	1.01 [0.99, 1.03]	0.263	-0.03 (0.00)	0.97 [0.97, 0.98]	0.000
IMD decile	-0.15 (0.04)	0.86 [0.80, 0.93]	0.000	-0.10 (0.04)	0.90 [0.83, 0.98]	0.015	0.15 (0.10)	1.16 [0.96, 1.41]	0.129	-0.07 (0.05)	0.93 [0.84, 1.04]	0.193
Study sub-period (c.f. Sub-period 1)												
Sub-period 2	0.07 (0.15)	1.07 [0.80, 1.44]	0.643	0.16 (0.21)	1.17 [0.78, 1.77]	0.444	-0.42 (0.32)	0.66 [0.36, 1.22]	0.185	0.30 (0.24)	1.34 [0.84, 2.15]	0.219
Sub-period 3	0.24 (0.14)	1.27 [0.96, 1.68]	0.094	0.09 (0.18)	1.09 [0.77, 1.54]	0.620	-0.26 (0.20)	0.77 [0.52, 1.14]	0.193	0.18 (0.24)	1.19 [0.74, 1.91]	0.466
Sub-period 4	0.25 (0.18)	1.29 [0.91, 1.81]	0.151	0.16 (0.20)	1.17 [0.79, 1.74]	0.428	0.21 (0.20)	1.24 [0.84, 1.81]	0.282	0.34 (0.23)	1.41 [0.91, 2.19]	0.130
Sub-period 5	0.22 (0.18)	1.24 [0.87, 1.77]	0.232	0.15 (0.19)	1.17 [0.81, 1.68]	0.417	0.04 (0.25)	1.04 [0.64, 1.71]	0.867	0.24 (0.22)	1.28 [0.84, 1.95]	0.256
History of anxiety	0.23 (0.10)	1.26 [1.04, 1.53]	0.017	-0.12 (0.14)	0.89 [0.68, 1.15]	0.371	-0.25 (0.14)	0.78 [0.59, 1.03]	0.076	-0.33 (0.16)	0.72 [0.52, 0.99]	0.041

History of depression	0.39 (0.14)	1.48 [1.13, 1.93]	0.004	-0.10 (0.11)	0.91 [0.74, 1.12]	0.362	-0.31 (0.10)	0.73 [0.60, 0.90]	0.003	-0.31 (0.14)	0.73 [0.55, 0.97]	0.031
History of SMI	2.85 (0.15)	17.31 [12.88, 23.26]	0.000	3.85 (0.20)	47.15 [32.14, 69.16]	0.000	3.73 (0.17)	41.71 [29.9, 58.19]	0.000	4.35 (0.27)	77.16 [45.44, 131.04]	0.000
Total GP consultations	0.07 (0.01)	1.08 [1.06, 1.09]	0.000	0.05 (0.01)	1.05 [1.03, 1.07]	0.000	0.03 (0.01)	1.03 [1.01, 1.06]	0.006	0.03 (0.01)	1.03 [1.00, 1.05]	0.076

CI: Confidence Interval; IMD: Index of Multiple Deprivation; IRR: Incidence Rate Ratio; MHA: Mental Health Act; SE: Standard Error; SMI: Severe Mental Illness.
All associations were estimated using Generalised Estimating Equations clustering by GP practice. Models included an offset for time at risk to account for varying follow-up durations.

Section E: Interactions

Supplementary Table 12: Interaction analyses between consultation modality and demographic characteristics for the primary analysis (MICE)

Interaction Term	Emergency contacts with mental health liaison teams		Psychiatric hospital admissions		Inpatient bed-days		MHA admissions	
	IRR [95% CI]	P value	IRR [95% CI]	P value	IRR [95% CI]	P value	IRR [95% CI]	P value
Proportion of remote consultations x Age	1.00 [1.00, 1.00]	0.742	1.00 [1.00, 1.00]	0.208	1.00 [1.00, 1.00]	0.498	1.00 [1.00, 1.00]	0.059
Proportion of remote consultations x Sex (Female)	1.03 [0.97, 1.10]	0.293	1.03 [0.96, 1.10]	0.404	1.12 [1.02, 1.22]	0.019	1.03 [0.95, 1.11]	0.496
Proportion of remote consultations x Ethnicity								
Black, Black British, Caribbean or African	1.00 [0.94, 1.07]	0.985	1.00 [0.94, 1.06]	0.904	1.06 [0.97, 1.16]	0.177	1.06 [0.86, 1.31]	0.632
Asian or Asian British	0.99 [0.84, 1.16]	0.877	1.04 [0.89, 1.22]	0.587	1.000 [0.79, 1.27]	0.991	1.02 [0.94, 1.10]	0.586
Mixed or multiple ethnic groups	0.99 [0.94, 1.06]	0.818	1.03 [0.94, 1.12]	0.526	1.03 [0.92, 1.16]	0.596	1.06 [0.94, 1.19]	0.352
Other ethnic group	1.04 [0.84, 1.28]	0.720	1.01 [0.82, 1.23]	0.961	1.08 [0.85, 1.38]	0.545	1.01 [0.74, 1.37]	0.964
Proportion of remote consultations x IMD decile	1.01 [0.99, 1.03]	0.350	1.01 [0.10, 1.03]	0.182	1.00 [0.96, 1.04]	0.900	1.02 [1.00, 1.04]	0.086

Supplementary Table 13: Interaction analyses between consultation modality and demographic characteristics for complete case analysis

Interaction	Emergency contacts with mental health liaison teams		Psychiatric hospital admissions		Inpatient bed-days		MHA admissions	
	IRR [95% CI]	P value	IRR [95% CI]	P value	IRR [95% CI]	P value	IRR [95% CI]	P value
Proportion of remote consultations x Age	1.00 [1.00, 1.00]	0.515	1.00 [1.00, 1.00]	0.221	1.00 [0.99, 1.00]	0.468	1.00 [1.00, 1.00]	0.067
Proportion of remote consultations x Sex (Male)	1.06 [1.00, 1.13]	0.070	1.04 [0.97, 1.12]	0.234	1.10 [1.00, 1.22]	0.056	1.03 [0.95, 1.12]	0.422
Proportion of remote consultations x Ethnicity								
Black, Black British, Caribbean or African	1.01 [0.94, 1.09]	0.748	0.99 [0.93, 1.06]	0.767	1.05 [0.97, 1.15]	0.228	1.02 [0.94, 1.10]	0.622
Asian or Asian British	0.95 [0.82, 1.11]	0.502	1.00 [0.83, 1.20]	0.987	0.90 [0.77, 1.05]	0.187	1.02 [0.83, 1.24]	0.886
Mixed or multiple ethnic groups	1.01 [0.94, 1.07]	0.880	1.03 [0.95, 1.12]	0.509	1.03 [0.94, 1.11]	0.560	1.07 [0.95, 1.20]	0.251
Other ethnic group	1.10 [0.91, 1.31]	0.334	1.02 [0.85, 1.22]	0.868	1.05 [0.88, 1.25]	0.563	1.02 [0.76, 1.38]	0.896
Proportion of remote consultations x IMD decile	1.01 [0.99, 1.03]	0.336	1.01 [1.00, 1.03]	0.155	1.01 [0.97, 1.05]	0.727	1.02 [1.00, 1.04]	0.101

IRR: Incidence Rate Ratio; CI: Confidence Interval; IMD: Index of Multiple Deprivation; MHA: Mental Health Act.
Values represent the interaction term (ratio of ratios).
