# Supplementary material for: Primary care consultation modality and acute mental health service use in adults
Source: Nat Ment Health. 2026 Mar 17;4(4):574–81. doi: 10.1038/s44220-026-00605-9 (PMC13076198; doi:10.1038/s44220-026-00605-9)
Supplement: Supplementary file 2 — Reporting Summary [file 44220_2026_605_MOESM2_ESM.pdf]

nature portfolio

Corresponding author(s): Mariana Pinto da Costa

Last updated by author(s): Jan 16, 2026

Reporting Summary

Nature Portfolio wishes to improve the reproducibility of the work that we publish. This form provides structure for consistency and transparency in reporting. For further information on Nature Portfolio policies, see our Editorial Policies and the Editorial Policy Checklist.

Statistics

For all statistical analyses, confirm that the following items are present in the figure legend, table legend, main text, or Methods section.

n/a	Confirmed	
☐	☒	The exact sample size (*n*) for each experimental group/condition, given as a discrete number and unit of measurement
☐	☒	A statement on whether measurements were taken from distinct samples or whether the same sample was measured repeatedly
☐	☒	The statistical test(s) used AND whether they are one- or two-sided<br>*Only common tests should be described solely by name; describe more complex techniques in the Methods section.*
☐	☒	A description of all covariates tested
☐	☒	A description of any assumptions or corrections, such as tests of normality and adjustment for multiple comparisons
☐	☒	A full description of the statistical parameters including central tendency (e.g. means) or other basic estimates (e.g. regression coefficient) AND variation (e.g. standard deviation) or associated estimates of uncertainty (e.g. confidence intervals)
☒	☐	For null hypothesis testing, the test statistic (e.g. *F*, *t*, *r*) with confidence intervals, effect sizes, degrees of freedom and *P* value noted<br>*Give P values as exact values whenever suitable.*
☒	☐	For Bayesian analysis, information on the choice of priors and Markov chain Monte Carlo settings
☐	☒	For hierarchical and complex designs, identification of the appropriate level for tests and full reporting of outcomes
☐	☒	Estimates of effect sizes (e.g. Cohen's *d*, Pearson's *r*), indicating how they were calculated

Our web collection on statistics for biologists contains articles on many of the points above.

Software and code

Policy information about availability of computer code

Data collection	The Clinical Record Interactive System (CRIS) and its linkage to Lambeth DataNet (LDN), developed by the NIHR Maudsley Biomedical Research Centre (BRC) at the South London and Maudsley NHS Foundation Trust was used to retrieve data.
Data analysis	Analytic scripts used to generate the final results presented in the manuscript, including data cleaning and analysis, have been deposited in the Zenodo repository under the identifier: https://doi.org/10.5281/zenodo.17949821<br>avantgarda/gp-consultation-modality-mental-health: v1.0.0

For manuscripts utilizing custom algorithms or software that are central to the research but not yet described in published literature, software must be made available to editors and reviewers. We strongly encourage code deposition in a community repository (e.g. GitHub). See the Nature Portfolio guidelines for submitting code & software for further information.

Data

Policy information about availability of data

All manuscripts must include a data availability statement. This statement should provide the following information, where applicable:

- Accession codes, unique identifiers, or web links for publicly available datasets
- A description of any restrictions on data availability
- For clinical datasets or third party data, please ensure that the statement adheres to our policy

The data that support the findings of this study are pseudonymised electronic health records from the South London and Maudsley NHS Foundation Trust (CRIS)

and Lambeth DataNet. Due to ethical and information governance restrictions regarding patient confidentiality, these data are not publicly available. Access to the data is restricted to researchers with appropriate honorary contracts and approvals. Information regarding the CRIS system and the formal application procedures required for external researchers can be found at: https://www.maudsleybrc.nihr.ac.uk/facilities/clinical-record-interactive-search-cris/

Research involving human participants, their data, or biological material

Policy information about studies with human participants or human data. See also policy information about sex, gender (identity/presentation), and sexual orientation and race, ethnicity and racism.

Reporting on sex and gender	The study used the sex data available in the electronic health records of the CRIS database. Sex is based on self-report and recorded by NHS staff. The sample included 40.2% of males and 59.8% of females.
Reporting on race, ethnicity, or other socially relevant groupings	The study used the sex data available in the electronic health records of the CRIS database. Ethnicity is based on self-report and recorded by NHS staff using categories specified by the NHS. Ethnicity categories in the sample were: White (45.7%), Black, Black British, Caribbean or African (21.5%), Asian or Asian British (7.3%), Mixed or multiple ethnic groups (22.8%), Other ethnic groups (2.8%).
Population characteristics	See below.
Recruitment	This study analysed pseudonymised data from electronic health records from a linkage between the CRIS database at South London and Maudsley NNHS Foundation Trust and LDN between 2019 and 2021.
Ethics oversight	The Clinical Record Interactive Search database and its linkage with Lambeth DataNet have ethical approval from the South Central Oxford C Research Ethics Committee (REC) for use as a secondary research database (REC reference: 23/SC/0257). This study received approval by CRIS (reference: 22-040) on 8 June 2022 (including external data linkages).

Note that full information on the approval of the study protocol must also be provided in the manuscript.

Field-specific reporting

Please select the one below that is the best fit for your research. If you are not sure, read the appropriate sections before making your selection.

☐ Life sciences ☒ Behavioural & social sciences ☐ Ecological, evolutionary & environmental sciences

For a reference copy of the document with all sections, see nature.com/documents/nr-reporting-summary-flat.pdf

Behavioural & social sciences study design

All studies must disclose on these points even when the disclosure is negative.

Study description	This is a quantitative retrospective cohort study using an exposure-outcome design investigating the relationship between primary care consultation modalities and acute mental health service use.
Research sample	We included all patients registered with a primary care provider in the London borough of Lambeth within the study period who had a diagnosis of anxiety, depression or SMI, including bipolar and psychotic disorders, according to the Systematized Nomenclature of Medicine Clinical Terms (SNOMED), a structured clinical vocabulary used in electronic health records. This approach ensured consistent identification of clinicla diagnoses across electronic health record systems. Patients were excluded if they were under 18 years of age at exposure, were not registered to a GP or received zero consultations during the exposure period, or died before the start of the outcome period.<br>The study sample was drawn from a linkage between LDN, which captures routinely collected primary care data for all general practices in the borough, and the CRIS database from the South London and Maudsley NHS Foundation Trust, which provides detailed secondary mental healthcare records. These datasets are widely used for local population health research and together offer comprehensive coverage of residents who engage with both GP and specialist mental health services. The combined dataset is therefore broadly representative of adults with common mental disorders and SMI living in an urban, ethnically diverse, and socioeconomically varied inner-London population.<br>Sex and ethnicity information were obtained from the electronic health records. Sex reflects the recorded administrative category within CRIS. Ethnicity is self-reported and recorded by NHS staff using standard NHS ethnicity categories. In our sample, the distribution of ethnicity was: White (45.7%), Black, Black British, Caribbean or African (21.5%), Asian or Asian British (7.3%), Mixed or multiple ethnic groups (22.8%), and Other ethnic groups (2.8%). These proportions reflect the diverse demographic profile of Lambeth.<br>Our rationale for selecting this study population was to investigate real-world consultation patterns among adults with mental health conditions in a setting where both primary care and specialist mental health services are comprehensively captured through linked records.
Sampling strategy	The study period was divided into five exposure-outcome sub-periods, equally distributed over the full timeline. Each exposure period was of six months in length. We calculated 'active days', defined as the number of days the patient was registered with a GP during this window. The exposure metric was the proportion of remote GP consultations experienced during that time, which was calculated as the number of remote GP consultations divided by the total number of GP consultations with valid (non-missing) modalities. At the end of each exposure sub-period, the outcome measures were then calculated for each patient in the subsequent six-month outcome period, adjusting for 'days at risk', which restricted the number of follow-up days if the patient died during the outcome phase.

	The data from each exposure-outcome sub-period were then recombined. If a patient featured in more than one eligible sub-period, only one set of their data was used in the recombination, selected by random.
Data collection	Data for this study were obtained from routinely collected electronic health records through a linkage between LDN and the CRIS system. LDN captures structured primary care data recorded by general practitioners using standardised clinical coding systems, while CRIS extracts de-identified secondary mental healthcare records from the South London and Maudsley NHS Foundation Trust's electronic patient record system. No direct researcher–participant interaction occurred, and no individuals other than clinical staff were present at the time data were originally entered into the records as part of routine care. No experimental manipulation took place and there were no experimental conditions to which blinding could apply.
Timing	01-01-2019 – 31/12/2021
Data exclusions	We initially identified 110,310 patients meeting inclusion criteria. We excluded 2,317 patients due to meeting pre-established exclusion criteria as described above.
Non-participation	No participants dropped out due to using administrative data.
Randomization	Participants were not allocated to experimental groups.

Reporting for specific materials, systems and methods

We require information from authors about some types of materials, experimental systems and methods used in many studies. Here, indicate whether each material, system or method listed is relevant to your study. If you are not sure if a list item applies to your research, read the appropriate section before selecting a response.

Materials & experimental systems

n/a	Involved in the study
☒	☐ Antibodies
☒	☐ Eukaryotic cell lines
☒	☐ Palaeontology and archaeology
☒	☐ Animals and other organisms
☒	☐ Clinical data
☒	☐ Dual use research of concern
☒	☐ Plants

Methods

n/a	Involved in the study
☒	☐ ChIP-seq
☒	☐ Flow cytometry
☒	☐ MRI-based neuroimaging

Plants

Seed stocks	*Report on the source of all seed stocks or other plant material used. If applicable, state the seed stock centre and catalogue number. If plant specimens were collected from the field, describe the collection location, date and sampling procedures.*
Novel plant genotypes	*Describe the methods by which all novel plant genotypes were produced. This includes those generated by transgenic approaches, gene editing, chemical/radiation-based mutagenesis and hybridization. For transgenic lines, describe the transformation method, the number of independent lines analyzed and the generation upon which experiments were performed. For gene-edited lines, describe the editor used, the endogenous sequence targeted for editing, the targeting guide RNA sequence (if applicable) and how the editor was applied.*
Authentication	*Describe any authentication procedures for each seed stock used or novel genotype generated. Describe any experiments used to assess the effect of a mutation and, where applicable, how potential secondary effects (e.g. second site T-DNA insertions, mosiacism, off-target gene editing) were examined.*
